# Potential Toxicological Risk Associated with the Oral Use of Colloidal Silver Dietary Supplements

**DOI:** 10.3390/toxics13110992

**Published:** 2025-11-18

**Authors:** Oana Catalina Bute, Anca-Irina Gheboianu, Bogdan Trica, Ana-Maria Hossu

**Affiliations:** 1 Faculty of Sciences and Arts, Valahia University of Târgoviște, 13 Sinaia Alley, 130004 Târgoviște, Romania; oana.bute@valahia.ro; 2 Institute of Multidisciplinary Research for Science and Technology, Valahia University of Târgoviște, 13 Sinaia Alley, 130004 Târgoviște, Romania; 3 National Institute for Research & Development in Chemistry and Petrochemistry—ICECHIM Bucharest, 202 Splaiul Independentei, 060021 Bucharest, Romania; trica.bogdan@gmail.com

**Keywords:** colloidal silver, dietary supplements, nanoparticles, oral exposure, estimated toxicological risk

## Abstract

The increasing availability of colloidal silver dietary supplements raises important concerns regarding their safety when used for oral consumption. This study presents the physicochemical characterization of a commercial colloidal silver solution with a high concentration (1000 mg/L), stabilized with pectin in distilled water. The characterization was performed using UV-VIS, XRD, and TEM. The manufacturer did not provide information regarding nanoparticle size or recommended duration of use. The 1000 mg/L sample was also compared with a standard colloidal silver solution provided by Sigma Aldrich to validate nanoparticle size, stability, and spectral profiles. In addition, a comparative theoretical analysis was conducted on other commercially available products containing colloidal silver at concentrations of 15, 30, 55, 80, and 125 mg/L, based solely on the recommended daily intake and the oral reference dose of 0.005 mg/kg/day established by the United States Environmental Protection Agency (EPA). Although no in vivo or in vitro toxicity tests were performed, the results indicate a potential toxicological risk due to estimated intake levels that may exceed safety thresholds, particularly in high-concentration products with insufficient labelling or unclear usage guidelines. These findings emphasize the need for stricter regulatory measures and greater public awareness regarding the internal use of colloidal silver supplements.

## 1. Introduction

The use of colloidal silver dietary supplements pertains largely to the field of alternative medicine, which lacks robust scientific evidence supporting its efficacy or safety [1]. Despite being marketed as natural remedies with purported antimicrobial, immune-boosting, and detoxifying properties, these claims are not validated by controlled clinical trials [2]. Historically, silver compounds were used as antiseptics before the introduction of modern antibiotics [3], but their uncontrolled internal administration is associated with toxic effects, most notably argyria—a permanent bluish-grey discoloration of the skin and mucous membranes due to silver deposition in tissues [4].

Recently, colloidal silver products have regained popularity among consumers seeking alternative treatments, despite repeated warnings from health authorities. Agencies such as U.S. Food and Drug Administration (FDA), the European Chemicals Agency (ECHA), the World Health Organization (WHO), and national agencies such as Valvira in Finland have issued public statements discouraging oral use, citing, the absence of demonstrated therapeutic benefits and the potential for cumulative toxicity [5,6,7,8].

Scientific studies have documented a variety of adverse effects linked to prolonged exposure to silver nanoparticles, including bioaccumulation in internal organs such as the liver, spleen, kidneys, and brain, as well as oxidative stress, mitochondrial dysfunctions, DNA damage, and gene expression alterations [9,10,11,12]. Of particular concern are the potential reproductive effects observed in animal models, with nanoparticles shown to cross the placental barrier and interfere with fetal development [13,14]. Such effects are dose and time dependent, and even low daily doses exceeding established safety thresholds such as the 0.005 mg/kg/day reference dose proposed by U.S. Environmental Protection Agency (EPA) may lead to long term silver accumulation in tissues [15].

This study investigates the potential health risks associated with the oral use of colloidal silver dietary supplements marketed in Romania. The products with the highest silver concentration (1000 mg/L) stabilized with pectin in distilled water was selected for physicochemical characterization. The choice was based on the high silver concentration and on the lack of information provided by the manufacturer regarding nanoparticle size and recommended exposure duration. The product was supplied by a Romanian manufacturer company.

Characterization was performed using UV-VIS spectrophotometry, X-ray diffraction (XRD) and transmission electron microscopy (TEM). A certified colloidal silver standard (20 mg/L, 10 nm, sodium citrate stabilized in distilled water), obtained from Sigma-Aldrich, was used for comparison. The reference was employed to confirm the presence of surface plasma resonance band and to evaluate nanoparticle size and dispersion.

In addition, five other commercial supplements with lower silver concentration (15 mg/L, 30 mg/L, 55 mg/L, 80 mg/L, and 125 mg/L) were considered. These products were provided by the same domestic company and were analysed in relation to the oral reference dose established by the U.S. Environmental Protection Agency (0.005 mg/kg/day). The aim of this work was to estimate the potential risk associated with daily intake.

## 2. Materials and Methods

### 2.1. Sample Description

This study analysed a series of colloidal silver dietary supplements provided by a Romanian manufacturer. The primary sample, a commercially available product labelled to contain 1000 mg/L of silver nanoparticles, was stabilized with pectin in distilled water, as specified on the label. Due to its unusually high concentration and the absence of detailed information regarding nanoparticle size and recommended exposure duration, this sample was subjected to an in-depth physicochemical characterization. To support comparison, a certified colloidal silver reference solution from Sigma-Aldrich (Saint Louis, MO, USA) was used. This standard contains silver nanoparticles stabilized with sodium citrate in distilled water, with a concentration of 20 mg/L and a nominal particle diameter of 10 nm, as confirmed by TEM. The reference was employed to benchmark the optical and dispersion properties of the 1000 mg/L commercial sample.

In addition to the 1000 mg/L, five other supplements from the same manufacturer were included in the analysis. These products, with silver concentrations of 15, 30, 55, 80, and 125 mg/L, were all labelled as electrocolloidal silver stabilized in distilled water. According to the producer’s specifications, these formulations contain silver nanoparticles with diameters ranging from 0.5 to 5 nm. These lower concentrations samples were not characterized physicochemically. Their analysis focused on estimating potential toxicological exposure based on declared concentrations and recommended daily intake.

All samples were stored in accordance with the manufacturer’s instructions to maintain stability during the course of analytical procedures.

### 2.2. Analytical Methods

The characterization techniques used were UV-VIS spectroscopy (Shimadzu Corporation, Kyoto, Japan), X-ray diffraction (XRD) (Rigaku Corporation, Tokyo, Japan), and Transmission Electron Microscopy (TEM) (Thermo Fischer Scientific, Waltham, MA, USA) coupled with Energy Dispersive X-ray Spectroscopy (EDX) and Selected Area Electron Diffraction (SAED).

#### 2.2.1. UV-VIS Spectroscopy Method

UV-VIS spectroscopy was employed to optically characterize the metallic nanoparticles in suspension. Silver nanoparticles exhibit specific absorption due to Localized Surface Plasmon Resonance (LSPR), which manifests as a maximum absorption in the range of 390–450 nm depending on size, shape, aggregation, and stabilizer. Symmetric and well-defined spectra indicate uniform and dispersed particles, while broad or asymmetric bands may suggest aggregation or polydispersity. Measurements were performed using a Shimadzu UV-1900i spectrophotometer (Shimadzu Corporation, Kyoto, Japan) and quartz cuvettes (1 cm path length), scanning 300–700 nm. To avoid detector saturation and to optimise spectral resolution in the UV-VIS measurements, the 1000 mg/L colloidal silver solution was subjected to volumetric dilution using distilled water as the solvent. Three dilution ratios were prepared: 1:50, 1:100, and 1:200 (*v*/*v*). The reference sample was measured undiluted. pH measurements were performed for all colloidal samples to evaluate its influence on nanoparticle stability. pH affects surface charge, stabilizer ionization (sodium citrate, pectin), and electrostatic repulsion. Low pH can neutralize surface charge, promoting aggregation, while high pH can affect chemical stability. Optimal pH ranges depend on the specific stabilizer used.

#### 2.2.2. X-Ray Diffraction (XRD) Method

XRD analysis was performed using a Rigaku Ultima IV (Rigaku Corporation, Tokyo, Japan) diffractometer with a Cu Kα radiation (λ = 1.5406 Å), operating at 40 kV and 30 mA. Scans were carried out in continuous mode, high-resolution Bragg–Brentano, 2θ range 10–80°, step size 0.01°, and scan speed 1°/min. Data acquisition and processing were performed using PDXL 2.2 software together with the ICDD PDF 5+ 2024 database. Phase identification was carried out based on standard patterns from ICDD database, specifically using category S (Star Patterns—extremely high-quality data) and category I (Indexed Patterns—indexed high quality data). Samples were dried on glass slides in a conventional oven at 60 °C for 1 h to form uniform thin films. The resulting solid film was used directly for X-Ray diffraction measurements. This method allows detection of crystalline phases such as metallic silver (Ag^0^) with characteristic face-centered cubic (fcc) reflections, as well as secondary phases (oxides, salts, or synthesis byproducts), crystallite size, and structural parameters such as lattice constant, strain, and peak asymmetry.

#### 2.2.3. Transmission Electron Microscopy (TEM) Method

10 µL of dispersion were applied on a carbon film 300 mesh copper grid (TedPella, Redding, CA, USA). Excess was removed from the side using fine filter paper. The samples were left to dry and analysed using a Tecnai F20 G2 TWIN Cryo-TEM (Thermo Fischer Scientific, Waltham, MA, USA) in BF-TEM (Bright Field mode) at 200 kV. A selective Area Electron Diffraction (SAED) and an Energy Dispersive X-Ray Spectroscopy (EDS) system were included. These techniques were used to enable the direct observation of nanoparticle morphology and size distribution, as well as the assessment of crystalline characteristics (via electron diffraction) and elemental composition (via EDX). Digital Micrograph software version 2.12 (Gatan Inc.) was used for image acquisition, while TEM Imagining & Analysis (TIA) version 4.6.4 was employed for EDX spectral analysis.

## 3. Results

### 3.1. UV-VIS Spectroscopy Results

**Figure 1:**
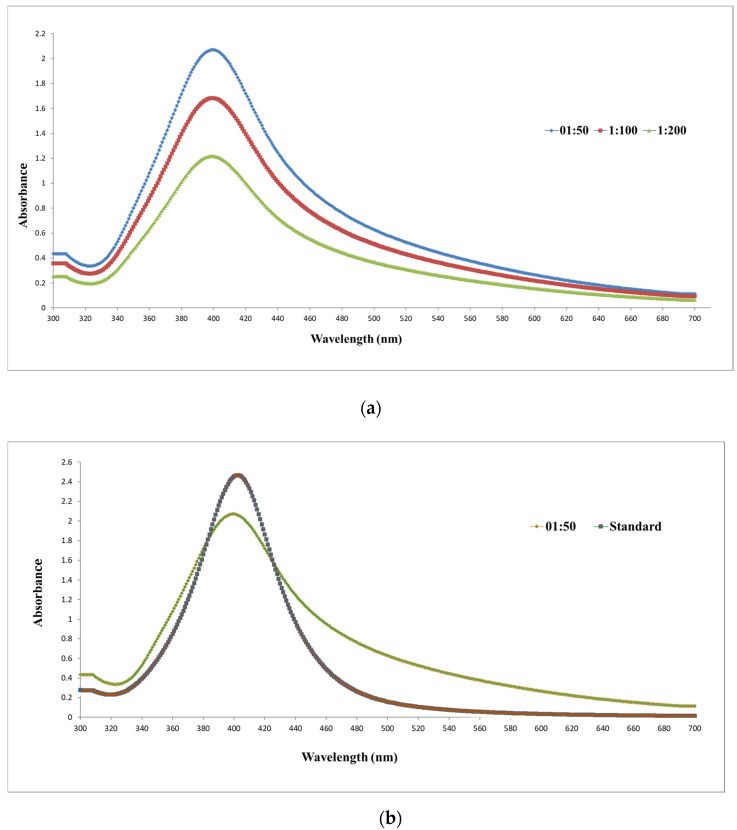
(**a**) UV-VIS spectra of the 1000 mg/L colloidal silver solution at dilutions of 1:50, 1:100, and 1:200. The optical densities at LSPR peak (400 nm) were 2.071, 1.684, and 1.217, respectively, confirming the proportional decrease with dilution and indicating colloidal stability. (**b**) Comparative UV-VIS spectra of standard colloidal silver solution (Sigma-Aldrich) containing nanoparticles of 10 nm in diameter at a concentration of 20 mg/L and of the 1:50 dilution of 1000 mg/L colloidal silver solution, highlighting differences in spectral profile and peak symmetry.

UV–VIS spectroscopy was applied to evaluate the optical properties of colloidal silver solutions and to verify the presence of local surface plasmon resonance (LSPR), which reflects nanoparticle size and dispersion. The focus was on a highly concentrated 1000 mg/L colloidal silver solution stabilized with pectin, compared to reference Sigma-Aldrich solution containing 10 nm silver nanoparticles stabilized with sodium citrate in distilled water (20 mg/L). Figure 1 presents the UV-VIS spectra.

Analysis of the recorded spectra clearly indicates the presence of a Local Surface Plasmon Resonance (LSPR) band at 400 nm, confirming the existence of silver nanoparticles in the colloidal solution. The optical densities were 2.071, 1.684, and 1.217 for the 1:50, 1:100, and 1:200 dilutions, respectively. Importantly, all three dilutions exhibited the same plasmon maximum at 400 nm, indicating that the nanoparticle system remained stable upon dilution (Figure 1a). The spectra were broader and asymmetrically extended toward longer wavelengths, suggesting a wider particle size distribution. When comparing the 1:50 dilution of the 1000 mg/L solution (equivalent to ~20 mg/L) with the standard Sigma-Aldrich solution of 20 mg/L, clear differences were observed (Figure 1b). Despite similar optical densities (2.071 vs. 2.468 for 10 nm), the pectin-stabilized colloid exhibited a broader, slightly blue-shifted peak at 400 nm, whereas the citrate-stabilized standards displayed narrow, symmetric LSPR peaks at 402 nm, typical for well-dispersed, monodisperse nanoparticles. A visual difference was also evident: the standard solutions appeared pale yellow, typical for stable silver nanoparticles of 10–20 nm at moderate concentrations, while the 1:50 dilution of the 1000 mg/L solution showed a slightly orange hue. This subtle colour shift may result from a wider size distribution including slightly larger particles, mild aggregation induced by pectin-based steric stabilization, or residual effects of the high initial nanoparticle concentration, which can lead to plasmonic coupling effects not observed in the monodisperse reference colloids. This contrast highlights the influence of the stabilizing agent: sodium citrate provides strong electrostatic stabilization, producing highly uniform nanoparticles, while pectin introduces steric stabilization, resulting in slightly more heterogeneous particles [16,17]. Long-term pH measurements over three months indicated stable alkaline conditions: 8.8 for the pectin-stabilized colloid compared to 9.0 for the citrate-stabilized standards, suggesting that both systems maintained colloidal stability over time despite different electrochemical environments. In summary, the UV-VIS spectra obtained for the three dilutions of the 1000 mg/L colloidal silver solution exhibit slight asymmetry and broadening, indicating that the system is polydisperse and composed of nanoparticles with a range of sizes compared to the Sigma-Aldrich reference.

### 3.2. X-Ray Diffraction (XRD) Results

**Figure 2:**
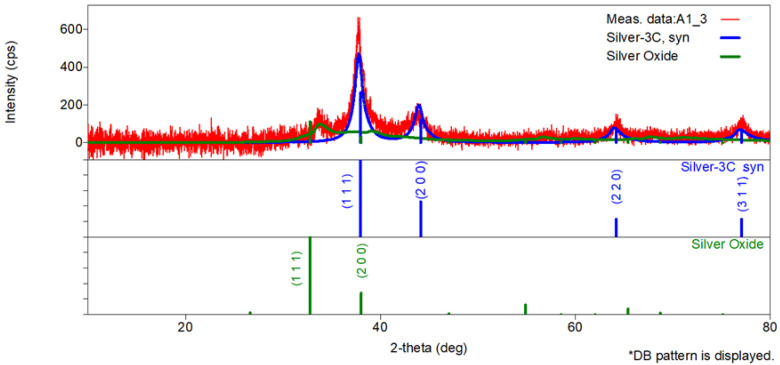
X-ray diffraction (XRD) pattern of the dried 1000 mg/L colloidal silver solution (A1_3). * DB—Powder Data Files DataBase pattern is displayed.

Figure 2 shows X-ray diffraction (XRD) pattern of the dried 1000 mg/L colloidal silver solution (the analysed sample was named A1_3).

The main diffraction peaks correspond to face-centered cubic metallic silver (Ag, JCPDS No. 04-0783) with reflections at 2θ values of approximately 37.6°, 43.9°, 64.3°, and 77.0°, which match the (111), (200), (220), and (311) planes as well as to silver oxide (Ag_2_O, JCPDS No. 41-1104) with a characteristic peak at around 33.7° suggesting partial surface oxidation (Figure 2). It is important to note that the formation of silver oxide may have been partially induced during sample preparation for XRD, as drying the colloidal suspension exposes nanoparticles to atmospheric oxygen, facilitating superficial oxidation.

#### 3.2.1. Phase Identification

The X-ray diffraction (XRD) pattern of the colloidal silver sample reveals the presence of two distinct crystalline phases as identified using ICDD PDF-5 + database:

- Silver (Ag): face-centered cubic (fcc) structure, space group Fm-3m, ICDD 04-003-7118, 42% (estimated by scale factor);
- Silver Oxide (Ag_2_O): cubic structure, space group Pn-3m, ICDD 00-041-1104, 58% (estimated by scale factor).

Following the identification of the crystalline phases present in the sample, the analysis was further refined by evaluating the relative peak intensities of the metallic silver phase. Relative intensity refers to the height of each diffraction peak normalized to the most intense peak, which is conventionally set to 100% [18,19,20]. This parameter provides essential insight into the preferential orientation of the crystallites within the sample. For face-centered cubic silver, standard reference data (JCPDS File No. 04-0783) provides a set of expected relative intensities for the (111), (200), (220), and (311) planes (Figure 2). Deviations from this pattern in the experimental data may indicate texture effects, preferred crystal growth, or synthesis-related structural variations [19]. Table 1 presents the calculated relative intensities of the main peaks corresponding to the fcc phase of silver, along with the standard values provided by JCPDS File No. 04-0783.

The experimental relative intensities reveal a dominant (111) reflection, significantly stronger than other planes, suggesting a preferred orientation of the silver nanoparticles along the (111) crystallographic direction. The reduced intensity of the (200) and (220) peaks, compared to standard, further supports the presence of anisotropic growth and texture effects induced during nanoparticle synthesis and film formation [21,22]. The (311) peak matches the expected standard intensity, which validates the phase assignment.

#### 3.2.2. Comparison Between Experimental and Standard Diffraction Peaks

To validate the crystallographic assignment and assess the phase purity of the synthesized silver nanoparticles, the experimental 2θ values obtained from the XRD pattern were compared with standard reference values for face-centered cubic (fcc) metallic silver (JCPDS file No. 04-0783). The differences in diffraction angles (Δ2θ) were calculated, and the results are summarized in Table 2.

The comparison between the experimental and standard diffraction angles shows very small deviations (Δ2θ < 0.5°), indicating that the identified peaks correspond accurately to the expected crystallographic planes of metallic silver (fcc, JCPDS No. 04-0783). The observed shifts are minor and with acceptable instrumental error margins.

#### 3.2.3. Crystallite Size Estimation

The crystallite size of the silver nanoparticles was estimated using Debye–Scherrer Equation (1) [19,20,23]. This method is commonly applied for evaluating the size of coherent diffraction domains in nanomaterials, and it assumes a negligible lattice strain contribution.

(1) $$D=\frac{K\cdot \lambda }{\beta cos\theta }$$

where D is the crystallite size, K = 0.9 is the shape factor, λ = 0.15406 nm is the X-ray wavelength of Cu Kα radiation, β is the full width at half maximum (FWHM) in radians, and θ is the Bragg angle in radians. The calculations were applied to the main diffraction peaks corresponding to the (111), (200), (220), and (311) planes of face-centered cubic (fcc) silver (Figure 2). The obtain crystallite sizes ranged from 4.44 nm to 9.33 nm, with individual values summarized in Table 3.

The average crystallite size calculated from XRD data using Debye–Scherrer equation was 6.68 ± 2.55 nm. This result was obtained based on four diffraction peaks corresponding to the (111), (200), (220), and (311) planes of face-centered cubic silver (Figure 2).

#### 3.2.4. Crystallinity Index Estimation

The crystallinity index (*I_cry_*) is a useful parameter that compares the average physical particle size determined by Transmission Electron Microscopy (TEM) with the crystallite size calculated from X-ray diffraction (XRD) analysis. This comparison provides insights into the structural nature of nanoparticles, particularly the presence of polycrystalline domains within individual particles [18,19,20].

The crystallinity index is calculated using Equation (2):

(2) $$I_{cry}=\frac{D_{P}\left(TEM\right)}{D_{cry}\left(XRD\right)}$$

where I_cry_ is the crystallinity index, D_P_ (TEM) is the particle size obtained from TEM image (see Section 3.3), and D_cry_(XRD) is the average crystallite size calculated from the Debye–Scherrer equation.

In this study, the average particle size determined by TEM was 7.94 nm, while the average crystallite size determined by the Debye–Scherrer equation applied to the four main diffraction peaks was 6.68 nm. Thus, the crystallinity index was 1.18. An I_cry_ value close to 1 indicates that the particles are likely monocrystalline, whereas a value greater than 1 suggests a polycrystalline structure [19,21]. The result obtained, I_cry_ = 1.18 supports the conclusion that the silver nanoparticles in the analysed colloidal solution exhibit a polycrystalline nature.

The quality of the Rietveld refinement is typically assessed using the weighted profile R-factor (Rwp) and the goodness of fit (S), which is defined as the ratio between Rwp and the expected R-factor (Rexp). In this study, the obtained values were Rwp = 7.17% and S = 1.0751, indicating a very good agreement between the experimental and calculated diffraction profiles. A value of S close to 1 suggests an accurate model without significant unaccounted structural features or secondary phases. These criteria are consistent with established practices in crystallographic analysis [22].

### 3.3. Transmission Electron Microscopy (TEM) Results

**Figure 3:**
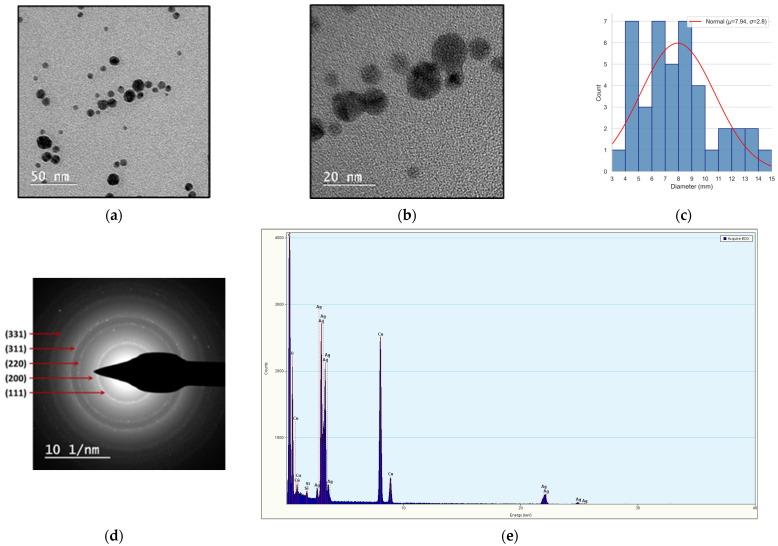
Representative BF-TEM micrographs, SAED pattern, and size distribution histogram of the silver nanoparticles: (**a**) Ag NPs at 100,000×; (**b**) Ag NPs at 280,000×; (**c**) size distribution histogram of the silver nanoparticles (raw data is in Table S1); (**d**) the concentric diffraction rings; (**e**) EDX spectrum.

In addition to UV-VIS spectroscopy and X-ray Diffraction (XRD), Transmission Electron Microscopy (TEM) was employed as a complementary analytical technique. When coupled with Energy Dispersive X-ray Spectroscopy (EDS) and Selected Area electron Diffraction (SAED), TEM enabled detailed investigation of the morphology, size distribution, and crystalline characteristics of the silver nanoparticles present in the colloidal solution (Figure 3).

TEM analysis showed that the silver nanoparticles exhibit predominantly spherical morphology, with minimal aggregation observed (Figure 3a,b). The size distribution histogram, derived from direct measurements of individual particles, indicates a moderately broad dispersion with diameters mainly in the 6–9 nm range (Figure 3c). The distribution is slightly skewed toward larger sizes, and calculated mean diameter was 7.94 nm, with a standard deviation of 2.8 nm. These results suggest variability in particle growth during synthesis [24].

Selected Area Electron Diffraction (SAED) analysis revealed the presence of the concentric diffraction rings, indicative of the polycrystalline structure [25]. The measured interplanar distances matched those expected for face-centered cubic (fcc) silver, confirming the crystallographic phase of the nanoparticles within the colloidal solution (Figure 3d). Table 4 presents the comparison between the experimentally measured ring-ratio values and standard reference values for face-centered cubic (fcc) along with the corresponding Miller indices.

The EDX spectrum acquired from the 1000 mg/L colloidal silver solution confirms the presence of elemental silver as the predominant component (Figure 3e). The detection of additional elements, such as copper and silicon, is attributed to the TEM grid substrate and potential contributions from the stabilizing agents or residual synthesis byproducts. The presence of the oxygen alongside silver in the EDX spectrum indicates surface oxidation, which is commonly observed in colloidal silver nanoparticles exposed to ambient conditions. The concurrent detection of Ag_2_O by XRD supports this interpretation (Figure 2).

## 4. Discussion

### 4.1. Comparison Between Estimated Daily Intakes and Regulatory Thresholds

Colloidal silver supplements marketed in Romania are available in various concentrations, including 15 mg/L, 30 mg/L, 55 mg/L, 80 mg/L, 125 mg/L and 1000 mg/L. All of the products originate from the same manufacturer. Among the commercially available formulations, the product containing 1000 mg/L silver represents the highest concentration identified in this survey. To contextualize the potential toxicological risk assessment, the recommended daily intake of colloidal silver supplements was compared with EPA reference dose. Although only the 1000 mg/L product was subjected to full physicochemical characterization, additional products with lower concentrations (15, 30, 55, 80 and 125 mg/L) were analysed in terms of their labelled silver content, recommended daily dose and frequency of intake.

The estimated daily intake of silver (mg/day) was calculated using the Formula (3):

daily intake (mg/day) = concentration (mg/mL) × volume per dose (mL) × frequency (doses/day),(3)

To assess potential health risks, the daily intake was normalized to body weight (mg/kg/day) using a reference adult weight of 70 kg (Formula (4)).

daily intake per body weight (mg/kg/day) = daily intake (mg/day) ÷ 70kg,(4)

The calculated daily silver exposure (mg/kg/day) for a 70 kg adult shows that even the lowest concentrations exceed the EPA reference dose (0.005 mg/kg/day) [15] when consumed at the upper recommended limits. Table 5 summarizes these findings.

As shown in Table 5, even the lowest-concentration products (15–30 mg/L) exceed the EPA reference dose (0.005 mg/kg/day) when consumed at the maximum recommended frequency. Higher concentrations (125 mg/L and especially 1000 mg/L) lead to daily exposures that surpass these thresholds by one to two orders of magnitude, highlighting a significant risk of chronic accumulation and potential systemic toxicity.

### 4.2. Toxicological Effects of Silver Nanoparticles

#### 4.2.1. Organ-Level Toxicity

Silver nanoparticles (AgNPs) exhibit organ-specific accumulation patterns that depend on particle size, dose, exposure duration, and administration route. In a 90-day subchronic oral study, Fischer 344 rats exposed to AgNPs (56 nm) showed liver and renal damage at doses ≥ 125 mg/kg/day. Histopathological analysis revealed bile-duct hyperplasia, hepatocellular degeneration, and fibrosis. The No Observed Adverse Effect Level (NOAEL) was determined at 30 mg/kg/day, and the LOAEL at 125 mg/kg/day [10].

Repeated oral dosing also caused inflammatory infiltrates in intestinal tissues (duodenum, ileum, colon) and elevated pro-inflammatory cytokines at 1–10 mg/kg/day over two weeks [27]. Neurotoxicity of silver nanoparticles has been studied in rats after repeated oral exposure, where the particles were able to reach the brain and alter neurotransmitter levels. In particular, dopamine and serotonin concentrations in several brain regions changed after 28–90 days of daily oral dosing, indicating that silver nanoparticles (14 nm) can interfere with neurochemical signalling and may pose a risk to the central nervous system during prolonged intake [28].

The route of exposure further modulates biodistribution: oral administration mainly targets the liver and spleen, inhalation results in retention in the lungs and secondary deposition in the brain, while intravenous or subcutaneous routes lead to a more uniform systemic distribution but prolonged hepatic and splenic retention [29]. These findings indicate that repeated or long-term exposure, even at low doses, can result in tissue-specific bioaccumulation and chronic toxicity.

#### 4.2.2. Cellular and Molecular Effects

Silver nanoparticles (AgNPs) induce multiple toxic effects in human cell lines, strongly depending on particle size, dose, and exposure time. In human bronchial epithelial cells (BEAS-2B), exposure to 20 nm AgNPs at 10–50 µg/mL for 24 h significantly increased reactive oxygen species (ROS) production, DNA strand breaks (γ-H2AX foci), and triggered apoptosis [30]. Loeschner K. et al. (2011) suggest that after 28 days of repeated oral exposure to 14 nm AgNPs in rats, nanosized granules of silver were detected in the ileal epithelium and lysosomes of macrophages, showing cellular uptake and localization in the gut wall [9].

Silver nanoparticles of 5 nm can readily cross the intestinal epithelial barrier, enter cells via endocytosis and localize in lysosomes and mitochondria, where they trigger reactive oxygen species (ROS) production [27].

These findings collectively demonstrate that smaller AgNPs are more cytotoxic due to their higher surface area and increased Ag^+^ ion release, which amplifies oxidative stress and genotoxicity in human cells. Citrate-coated AgNPs were found to be significantly more genotoxic than PVP-coated particles, demonstrating the role of surface chemistry in modulating toxicity [11].

#### 4.2.3. Effects on Fertility and Fetal Development

Several recent studies have raised concerns regarding the potential reproductive and developmental toxicity of silver nanoparticles (AgNPs). These concerns are especially relevant for dietary supplements containing colloidal silver, which are sometimes used over prolonged periods.

In an in vivo study by Mozafari et al. (2020), pregnant mice were administered AgNPs (70 nm) at a dose of 1 mg/kg/day orally from gestational day 1 to 14. The results indicated significant fetal developmental alterations, including craniofacial malformations (e.g., exencephaly), spinal deformities, and syndactyly. Histopathological examination revealed apoptosis in fetal brain tissue and hepatic degeneration [31].

Zhang J. et.al. (2021) demonstrated that maternal oral exposure to silver nanoparticle (20 nm) in pregnant mice significantly reduce fetal weight [32].

Although the experimental doses are higher than typical human exposure from supplements (e.g., 0.11–0.17 mg/kg/day based on 125–1000 mg/L colloidal silver), the observed effects at 1 mg/kg/day imply that chronic exposure, especially in sensitive populations like pregnant women, may pose a considerable risk.

The 1 mg/kg/day dose originates from controlled toxicological studies in mice, where dosing is expressed per body weight. For comparison with human exposure, this value can be converted using body surface area scaling and the corresponding interspecies K_m_ coefficients [33].

Human Equivalent Dose (HED) conversion formula is Formula (5):

HED (mg/kg) = Animal dose (mg/kg) × (K_m_ animal/K_m_ human), (5)

For example, a dose of 1 mg/kg/day in mice corresponds to a human equivalent dose (HED) of approximately 0.081 mg/kg/day, calculating using conversion Formula (5) with K_m_(mouse) = 3, and K_m_(human) = 37.

This converted value is not a recommended human intake, but a theoretical interspecies comparison [34]. Notably, the EPA chronic oral reference dose for silver is much lower [0.005 mg/kg/day], and no safe daily intake has been established for nanoscale silver [35].

### 4.3. Regulatory Framework

The increasing availability of colloidal silver supplements on the global market has raised substantial concerns regarding their safety, efficacy, and regulatory oversight. Although some products claim health benefits and long-term safety, such statements are not always supported by scientific evidence or approved by regulatory authorities. This section explores the current regulatory positions on the use of colloidal silver in dietary supplements, beginning with international agencies and continuing with national-level oversight in Romania.

#### 4.3.1. International Scientific Agency Opinions

Several international scientific and regulatory agencies have issued evaluations and official positions regarding the use of silver nanoparticles (AgNPs) in food supplements, raising concerns about safety, regulatory gaps, and misleading marketing practices.

The French Agency for Food, Environmental and Occupational Health & Safety (ANSES) has concluded that the toxicological data currently available for silver nanoparticles is insufficient to determine their safety for internal use. Consequently, silver nanoparticles are not authorized as ingredients in food supplements in France. ANSES also recommends improved consumer information, traceability of nanomaterials, and enhanced product characterization [36].

The German Institute for Risk Assessment (BfR) does not recommend the use of silver nanoparticles in food, dietary supplements, or consumer products intended for human contact, due to significant data gaps regarding their absorption, distribution, long-term toxicity, and potential to induce antimicrobial resistance [37]. In the European Union, the European Chemicals Agency (ECHA) [6] and the European Food Safety Authority (EFSA) [38] play pivotal roles in regulating the use of silver in consumer products. According to the EU Biocidal Products Regulation (EU BPR No 528/2012), colloidal silver is not authorized for internal use, and any health claims associated with such usage are strictly prohibited. The EFSA has not approved silver as a dietary supplement ingredient due to insufficient evidence regarding its safety. The Scientific Committee on Consumer Safety (SCCS), a body under the European Commission, reviewed the use of AgNPs in cosmetic products and concluded in 2018 that there was insufficient evidence to confirm their safety for oral or dermal use. This reinforces concerns about their unregulated inclusion in ingestible supplements [39]. In Finland, the National Supervisory Authority for Welfare and Health (Valvira), the Finnish Medicines Agency (Fimea), and the Finnish Institute for Health and Welfare (THL) have all issued public warnings about colloidal silver supplements, citing lack of efficacy and possible health risks. These agencies also addressed misleading health claims in online marketing [8]. The Therapeutic Goods Administration (TGA) of Australia does not recognize colloidal silver as an effective oral therapeutic and has issued alerts regarding its potential toxicity [40]. The United States Food and Drug Administration (FDA) has taken regulatory action against several companies marketing colloidal silver with unproven health claims. The FDA emphasizes that colloidal silver is not considered safe or effective for any condition and should not be consumed [5]. According to the National Centre for Complementary and Integrative Health (NCCIH), colloidal silver has not been proven to be safe or effective for treating any disease. Long-term use can lead to serious side effects such as argyria. Therefore, NCCIH strongly advises against the oral use of colloidal silver as a dietary supplement [41].

#### 4.3.2. National Regulation in Romania

In Romania, the primary institutions responsible for supervising food supplements are the National Agency for Medicines and Medical Devices of Romania (ANMDMR) [42] and the National Sanitary Veterinary and Food Safety Authority (ANSVSA) [43]. ANMDMR is in charge of monitoring the compliance of health claims and ingredients in supplements, while ANSVSA handles product safety, labelling, and market surveillance.

Although Romania is part of the European Union and must comply with EU legislation—including the Biocidal Products Regulation (Regulation (EU) No 528/2012) [6]—which does not authorize silver for internal use, colloidal silver supplements are still widely available on the Romanian market. Many of these products are marketed online or in health stores under the umbrella of ‘natural medicine’ or ‘alternative therapies’, often making unsubstantiated claims about their antimicrobial or immunostimulant properties.

The lack of explicit national bans or restrictions, combined with inconsistent enforcement and limited consumer awareness, creates a regulatory gap. This gap permits the continued sale of products that contravene EU-level safety recommendations.

In Romania, the legal framework governing dietary supplements is defined by Law No 56/2021 on Food Supplements and Order No 1809/2007 approving the Norms on Dietary Supplements, which transpose the relevant European Union Directive into national legislation [44,45]. While these regulations establish requirements for market placement, labelling, and consumer protection, they do not address specific authorization for oral use of colloidal silver. Therefore, there is a pressing need for enhanced consumer awareness and clear information regarding the potential risks associated with oral consumption of colloidal silver supplements, especially by products with high silver concentration (1000 mg/L).

### 4.4. Public Awareness and Misleading Claims

The widespread marketing of colloidal silver dietary supplements continues despite strong warnings from major health authorities, including the European Food Safety Authority (EFSA) and the U.S. Food and Drug Administration (FDA), which state that silver has no proven benefits in food supplements and may pose health risks [5,35,38]. Many consumers remain unaware of these regulatory positions, partly due to misleading claims made by manufacturers and distributors. Numerous websites in Romania actively promote colloidal silver dietary supplements through persuasive language and scientifically unsupported claims. Some platforms encourage consumers to choose products based on the nanoparticle size, emphasizing that smaller silver particles-often in the range of 0.5–5 nm-are a marker of higher product quality. These claims are frequently based on the assumption that smaller nanoparticles have superior biological activity, without acknowledging the associated toxicological risks. In some cases, websites acknowledge that orally ingested colloidal silver is not approved by the U.S. Food and Drug Administration (FDA) yet paradoxically continue to promote its systemic use. The purported mechanism of action is described as “highly effective and very simple”, with claimed antibacterial and antiviral properties allegedly achieved “without any side effects”. These platforms often state that silver nanoparticles are naturally eliminated from the body after exerting their antimicrobial effect-an assertion that contradicts findings related to bioaccumulation and tissue retention [9,10,46,47]. Additionally, misleading statements are made regarding the physiological necessity of silver, including claims that “reduced silver levels in the body lead to reduced health”, thus promoting regular silver intake to “restore balance”. Such marketing further reinforces the idea that colloidal silver is non-toxic if consumed at the manufacturer’s recommended dosage, despite evidence from scientific literature and international health agencies indicating otherwise [35,38,39,40,41]. In contrast, a few platforms adopt a more cautious approach and acknowledge potential health risks. According to the NCCIH, long-term and regular intake of colloidal silver may cause severe side effects such as argyria, systemic toxicity, interactions with antibiotics, and even poisoning [41]. However, such warnings are often downplayed or listed alongside unsubstantiated benefits, leading to consumer misinformation. Furthermore, analysis of product labels reveals that critical safety information is often missing. In some supplements, the particle size is not disclosed at all, despite its relevance to toxicity and biological distribution. Additionally, certain labels claim that colour changes in the solution do not affect its properties-an assertion that lacks scientific support and may mislead consumers. On the other hand, product labels often mention the structural form of the silver nanoparticles, with some supplements indicating a crystalline structure, while others claim an amorphous form. However, no explanation is provided regarding the implications of these structural characteristics for biological reactivity or toxicity. This lack of clarification may mislead consumers into underestimating potential health risks associated with amorphous nanoparticles, which, according to recent studies, can exhibit higher chemical reactivity and increased toxic potential compared to their crystalline counterparts [47,48,49]. Notably, the duration of safe use is also frequently omitted, leaving users without guidance on the risks associated with chronic exposure. Such discrepancies underscore the urgent need for consumer education campaigns and stricter enforcement of labelling standards. Greater transparency regarding nanoparticle size, structural features, concentration, potential risks, and usage limitations is essential to enable informed decision-making by consumers.

### 4.5. Structural Features of Silver Nanoparticles and Toxicological Implications

An important aspect highlighted in the labelling of silver-containing food supplements is the distinction between amorphous and crystalline nanoparticle structures. Products in the 15–125 mg/L concentration range, which are electro-colloidal and colourless, explicitly mention the presence of amorphous silver nanoparticles with sizes between 0.5–5 nm. In contrast, the highly concentrated 1000 mg/L product indicates a crystalline structure on the label which was confirmed by XRD and SAED techniques. Recent literature has emphasized that nanoparticle’s structure significantly affects biological interactions and toxicity profiles [49,50,51]. Crystalline silver nanoparticles tend to exhibit different dissolution dynamics and generate reactive oxygen species (ROS) at varying levels compared to their amorphous counterparts. Studies demonstrate that amorphous AgNPs, due to their higher surface reactivity, can induce more pronounced toxic effects, including oxidative stress and DNA damage, even at lower doses [49]. Furthermore, the surface chemistry and structural disorder of amorphous particles facilitate greater interactions with cellular membranes and subcellular components, increasing their bioactivity and potential toxicity [52,53]. Therefore, the structural state of AgNPs—often overlooked in public-facing product descriptions—should be clearly communicated on product labelling and considered a critical factor in toxicological risk assessment and regulatory decisions.

## 5. Conclusions

In summary, this study highlights critical toxicological concerns related to colloidal silver-based dietary supplements commercially available in Romania. Products are marketed in a wide concentration range (15–1000 mg/L) and often include claims regarding nanoparticle size and structure, yet without scientific context or toxicological warnings. Based on physicochemical characterization of the 1000 mg/L colloidal silver solution, the following conclusions were drawn: UV-VIS spectroscopy revealed a localized surface plasmon resonance (LSPR) peak around 400 nm, typical of silver nanoparticles. Compared to the standard solution, the spectrum appeared broader and slightly asymmetric, suggesting the presence of particles with a range of sizes. This was confirmed by TEM analysis, which showed nanoparticles with diameters between 3 and 15 nm, with an average size of 7.94 nm. The polycrystalline nature of the particles was demonstrated by both XRD and SAED analyses. The presence of silver was confirmed through EDX spectroscopy. Even lower-concentration products (15–125 mg/L), containing amorphous AgNPs sized between 0.5–5 nm (as indicated by manufacturers), exceed reference doses established by EPA (0.005 mg/kg/day), especially when consumed at maximum recommended levels. Another critical observation is the lack of clear guidance on usage duration, as well as the absence of toxicity warnings on the labels. Moreover, public websites and commercial materials frequently present misleading claims, promoting these supplements as safe and highly effective without acknowledging scientific and regulatory evidence of potential harm.

From a toxicological perspective, nanoparticle size, shape, surface chemistry, concentration, and exposure duration are key determinants of risks. Amorphous nanoparticles, from the electro-colloidal products (according to the manufacturer’s label), may be more reactive and thus more toxic than crystalline ones, due to greater surface reactivity and biological interaction potential. Given the oral administration route and the potential for systemic distribution and organ accumulation, these findings underscore the need for clearer labelling, better regulation, and public education. Even low-dose products may pose long-term health risks due to the enhanced permeability of ultrasmall nanoparticles and their potential to bypass biological barriers.

Regulatory agencies should enforce harmonized standards aligned with EU and international recommendations. Public health campaigns should also highlight the risks associated with chronic exposure to colloidal silver, particularly in nanoparticulate form.

## Figures and Tables

**Table 1:** Experimental peak heights and relative intensities of fcc silver compared to standard values (JCPDS File No. 04-0783).

Plane (hkl)	Height (cps)	Relative Intensity (%)	Standard Intensity (%)
(111)	369	100.0	100
(200)	80	21.7	42
(220)	48	13.0	22
(311)	42	11.4	11

**Table 2:** Comparison between experimental and standard diffraction angles (2θ) for face-centered cubic (fcc) silver.

Plane (hkl)	2θ Experimental (°)	2θ Standard (°)	Δ2θ (°)
(111)	37.66	38.11	−0.45
(200)	43.94	44.29	−0.35
(220)	64.33	64.44	−0.11
(311)	77.00	77.47	−0.47

**Table 3:** Parameters used for crystallite size calculation of silver nanoparticles using Debye–Scherrer equation.

Plane (hkl)	2θ (°)	FWHM (°)	θ (Rad)	β (Rad)	Crystallite Size (nm)
(111)	37.66	1.00	0.32865	0.01745	8.39
(200)	43.94	1.88	0.38345	0.03281	4.56
(220)	64.33	2.11	0.56139	0.03683	4.44
(311)	77.00	1.09	0.67195	0.01902	9.33

**Table 4:** The comparison between the experimentally measured ring-ratio values and standard reference values [26] for face-centered cubic (fcc) along with the corresponding Miller indices.

No. i	*d_i_/d*_1_ Measured	*d_i_/d*_1_ Standard	Miller Indices
1	1.000	1.000	(111)
2	0.864	0.866	(200)
3	0.607	0.612	(220)
4	0.515	0.522	(311)
5	0.390	0.397	(331)

**Table 5:** Daily silver intake from colloidal silver supplements marketed in Romania and its comparison with EPA safety limits.

Supplement	Concentration (mg/mL)	Particle Size (nm)	Single Dose (mL)	Daily Frequency	Daily Intake (mg/Day)	Daily Intake Per 70 kg (mg/kg/Day)	Compared to EPA Reference Dose 0.005 mg/kg/Day
15 mg/L	0.015	0.5–5 nm	5–15	1–4	0.07–0.9 *	0.001–0.013	Exceeds EPA
30 mg/L	0.030	0.5–5 nm	5–15	1–4	0.15–1.8	0.002–0.026	Exceeds EPA
55 mg/L	0.055	0.5–5 nm	5–15	1–4	0.28–3.3	0.004–0.047	Exceeds EPA
80 mg/L	0.080	0.5–5 nm	5–15	1–4	0.4–4.8	0.006–0.069	Exceeds EPA
125 mg/L	0.125	0.5–5 nm	5–15	1–4	0.63–7.5	0.009–0.110	Exceeds EPA
1000 mg/L	1.000	3–15 nm **	0.5–3	1–4	0.5–12	0.007–0.171	Exceeds EPA

* 0.07 mg/day = 0.015 mg/mL × 5 mL × 1 dose/day; 0.9 mg/day = 0.015 mg/mL × 15 mL × 4 doses/day. ** particle size measured by TEM.

## Data Availability

The data presented in this case study are not publicly available due to confidentiality restrictions.

## Supplementary Materials

The following supporting information can be downloaded at: https://www.mdpi.com/article/10.3390/toxics13110992/s1, Table S1: Data for the size distribution histogram of the silver nanoparticles.

## Abbreviations

The following abbreviations are used in this manuscript:

XRD	X-Ray Diffraction
TEM	Transmission Electron Microscopy
WHO	World Health Organization
FDA	U.S. Food and Drug Administration
EPA	Environmental Protection Agency
ECHA	European Chemicals Agency
DNA	deoxyribonucleic acid
EDX	Energy Dispersive X-ray Spectroscopy
SAED	Selected Area Electron Diffraction
LSPR	Localized Surface Plasmon Resonance
fcc	face-centered cubic
JCPDS	Joint Committee on Powder Diffraction Standards
ICDD	International Centre for Diffraction Data
NCCIH	National Centre for Complementary and Integrative Health
ANSVSA	National Sanitary Veterinary and Food Safety Authority
ANMDMR	National Agency for Medicines and Medical Devices of Romania
Fimea	Finnish Medicines Agency
THL	Finnish Institute for Health and Welfare
